# Surveillance Metrics and History of the COVID-19 Pandemic in Central Asia: Updated Epidemiological Assessment

**DOI:** 10.2196/52318

**Published:** 2024-08-28

**Authors:** Alexander L Lundberg, Egon A Ozer, Scott A Wu, Alan G Soetikno, Sarah B Welch, Yingxuan Liu, Robert J Havey, Robert L Murphy, Claudia Hawkins, Maryann Mason, Chad J Achenbach, Lori A Post

**Affiliations:** 1 Buehler Center for Health Policy and Economics Robert J. Havey, MD Institute for Global Health Northwestern University Chicago, IL United States; 2 Department of Emergency Medicine Feinberg School of Medicine Northwestern University Chicago, IL United States; 3 Department of Medicine, Division of Infectious Diseases Feinberg School of Medicine Northwestern University Chicago, IL United States; 4 Center for Pathogen Genomics and Microbial Evolution Robert J. Havey, MD Institute for Global Health Northwestern University Chicago, IL United States; 5 Feinberg School of Medicine Northwestern University Chicago, IL United States; 6 Robert J. Havey, MD Institute for Global Health Northwestern University Chicago, IL United States; 7 Department of Medicine, General Internal Medicine and Geriatrics Feinberg School of Medicine Northwestern University Chicago, IL United States; 8 Center for Global Communicable and Emerging Infectious Diseases Robert J. Havey, MD Institute for Global Health Northwestern University, Chicago, IL United States

**Keywords:** SARS-CoV-2, COVID-19, Central Asia, pandemic, surveillance, public health, COVID-19 transmission, speed, acceleration, deceleration, jerk, dynamic panel, generalized method of moments, GMM, Arellano-Bond, 7-day lag, Armenia, Azerbaijan, Cyprus, Faeroe Islands, Georgia, Gibraltar, Kazakhstan, Kosovo, Kyrgyzstan, Macedonia, Russia, Tajikistan Turkey, Turkmenistan, Uzbekistan

## Abstract

**Background:**

This study updates the COVID-19 pandemic surveillance in Central Asia we conducted during the first year of the pandemic by providing 2 additional years of data for the region. The historical context provided through additional data can inform regional preparedness and early responses to infectious outbreaks of either the SARS-CoV-2 virus or future pathogens in Central Asia.

**Objective:**

First, we aim to measure whether there was an expansion or contraction in the pandemic in Central Asia when the World Health Organization (WHO) declared the end of the public health emergency for the COVID-19 pandemic on May 5, 2023. Second, we use dynamic and genomic surveillance methods to describe the history of the pandemic in the region and situate the window of the WHO declaration within the broader history. Third, we aim to provide historical context for the course of the pandemic in Central Asia.

**Methods:**

Traditional surveillance metrics, including counts and rates of COVID-19 transmissions and deaths, and enhanced surveillance indicators, including speed, acceleration, jerk, and persistence, were used to measure shifts in the pandemic. To identify the appearance and duration of variants of concern, we used data on sequenced SARS-CoV-2 variants from the Global Initiative on Sharing All Influenza Data (GISAID). We used Nextclade nomenclature to collect clade designations from sequences and Pangolin nomenclature for lineage designations of SARS-CoV-2. Finally, we conducted a 1-sided *t* test to determine whether regional speed was greater than an outbreak threshold of 10. We ran the test iteratively with 6 months of data across the sample period.

**Results:**

Speed for the region had remained below the outbreak threshold for 7 months by the time of the WHO declaration. Acceleration and jerk were also low and stable. Although the 1- and 7-day persistence coefficients remained statistically significant, the coefficients were relatively small in magnitude (0.125 and 0.347, respectively). Furthermore, the shift parameters for either of the 2 most recent weeks around May 5, 2023, were both significant and negative, meaning the clustering effect of new COVID-19 cases became even smaller in the 2 weeks around the WHO declaration. From December 2021 onward, Omicron was the predominant variant of concern in sequenced viral samples. The rolling *t* test of speed equal to 10 became entirely insignificant for the first time in March 2023.

**Conclusions:**

Although COVID-19 continues to circulate in Central Asia, the rate of transmission remained well below the threshold of an outbreak for 7 months ahead of the WHO declaration. COVID-19 appeared to be endemic in the region and no longer reached the threshold of a pandemic. Both standard and enhanced surveillance metrics suggest the pandemic had ended by the time of the WHO declaration.

## Introduction

### Background

COVID-19, the disease caused by the virus SARS-CoV-2, was first detected in Wuhan, China, in the fall of 2019 [1-5]. The first case of COVID-19 in Central Asia [6] is believed to have occurred in Russia on January 31, 2020 [7,8]. Our research team conducted an analysis of the pandemic in Central Asia 1 year into the pandemic [9]. This study provides 2 additional years of updated surveillance and analysis for the region.

We adopted the World Bank’s definition of Central Asia, which is based on economic development and geographical proximity, encompassing Armenia, Azerbaijan, Cyprus, Faeroe Islands, Georgia, Gibraltar, Kazakhstan, Kosovo, Kyrgyzstan, North Macedonia, Russia, Tajikistan, Turkey, Turkmenistan, and Uzbekistan [6].

The World Health Organization (WHO) declared the end of COVID-19 as a public health emergency of international concern on May 5, 2023 [10-12] based on the recommendation of the COVID-19 Emergency Committee [12]. To that end, we compared how the pandemic was progressing before and after the declaration.

### Empirical Definition of Pandemic Versus Epidemic Versus Outbreak Versus Endemic

Epidemiological terms, such as pandemic, epidemic, outbreak, and endemic, are used to describe the occurrence and spread of disease [13,14]. The distinctions between these terms lie in their scope, geographic extent, and severity. An epidemic refers to a sudden increase in the number of disease cases in a specific population or region. If the epidemic spreads across several countries or continents, it becomes a pandemic. An outbreak, on the other hand, describes a sudden increase in a concentrated setting, usually involving a more limited geographic area than an epidemic. Endemic refers to the constant presence of a disease in a particular geographic region or population, with no sudden increases in case volume [15,16].

### Traditional Surveillance Versus Enhanced Surveillance

Public health surveillance is the “ongoing, systematic collection, analysis, and interpretation of health-related data essential to planning and evaluation of public health practice” [17]. Surveillance not only explains the burden of death and disease due to a disease or a social condition but also generates research questions and guides researchers on topics that require further investigation [18-32]. Surveillance allows us to compare the burden of disease between geographical regions and to understand which regions are most impacted. The impact can be measured by rates of illness, hospitalization, and mortality and the associated economic costs of disease.

However, traditional surveillance metrics are often presented through either static or cumulative measures of infection rates and deaths [18-32]. In the middle of a burgeoning pandemic, policymakers and public health practitioners also need to use these metrics to understand what is about to happen. Is an outbreak increasing? Will growth switch from linear to exponential? Are more people dying from a condition in one place than another? To inform health policy and practice, knowledge of what is about to happen is often critical. Traditional surveillance metrics can be used to know and forecast infection rates and mortality. To that end, we developed enhanced surveillance metrics that translate traditional metrics into predictions of growth, including where along the epidemiological outbreak curve a particular region is situated. We also include metrics about the speed of the pandemic at the national, regional, and global levels. We measure how acceleration of speed this week compares with last week, as well as how novel infections last week predict new cases this week. We can think of the latter measure as the echoing forward of cases. These metrics were tested and validated in prior research [9,33-43]. For this study, we used both traditional and enhanced surveillance metrics to analyze the possible end to the pandemic in Central Asia.

### Objective

This study had 3 objectives. First, we aimed to measure whether there was an expansion or contraction in the pandemic in Central Asia when WHO declared the end of the COVID-19 pandemic as a public health emergency of international concern on May 5, 2023. At both the region and country levels, we used advanced surveillance techniques to describe the status of the pandemic in a 2-week window around the WHO declaration. From a public health perspective, we need to know whether the rate of new COVID-19 cases was increasing, decreasing, or stable from week to week and if any changes in the transmission rate indicated an acceleration or deceleration of the pandemic. Statistical insignificance is significant—it can signal the epidemiological “end” to the pandemic if the rate of new cases is 0 (or very low) and stable, meaning the number of new cases is neither accelerating nor decelerating.

Second, we used dynamic and genomic surveillance methods to describe the history of the pandemic in the region and situate the time window around the WHO declaration within the broader history. We included the ratio of COVID-19 deaths to the number of transmissions as a proxy for the mortality risk from infection at the population level. We also included a historical record of genomic surveillance from sequenced viral specimens to identify the appearance and spread of variants of concern in the region.

Third, we aimed to provide historical context for the course of the pandemic in Central Asia. We addressed several questions: How did countries respond to the pandemic? How did the region fare in terms of disease burden? In addition, what social, economic, and political factors shaped the course of COVID-19 in the region? This context can provide important lessons for disease prevention and mitigation in future pandemics.

## Methods

### Data Sources

This study conducted trend analyses with longitudinal COVID-19 data from Our World in Data (OWID) [44]. OWID compiles data on COVID-19 cases and mortality from various sources, including individual websites, statistical reports, and press releases. For the region of Central Asia, the data comprised an unbalanced panel of 16 countries and territories, running from July 31, 2020, to May 12, 2023. To analyze the pandemic over time, we used traditional and enhanced surveillance indicators. Traditional indicators include total cases and deaths, along with the 7-day moving average of new cases and deaths [9]. Enhanced surveillance metrics include (1) speed: the weekly average number of new positive tests per day per total country population multiplied by 100,000; (2) acceleration: the weekly average of the day-over-day changes in speed; (3) jerk: the week-over-week change in the acceleration rate of transmissions; and (4) 7-day persistence: the predictive effect of speed, indicating the number of new cases statistically attributable to new cases reported 7 days before. These transmission metrics can identify not only the presence and severity of outbreaks but also whether outbreaks are contracting, escalating, or imminent. For a full glossary of terms, see Multimedia Appendix 1.

To derive the 7-day persistence effect on speed, we established an empirical difference equation that links the number of new positive cases in each country on each day to the number of cases 7 days prior, weekend indicators, and weekly shift variables:

y*_it_*=ρy*_it_*_-1_+β**X***_it_*+α*_i_*+u*_it_* **(1)**

The dependent variable y_it_ is speed in country *i* at time *t*, and the independent variables **X***_it_* include weekend and recent week indicators, while α*_i_* denotes a country fixed effect and u*_it_* is the idiosyncratic error term. Please see the initial study for more details [9]. We estimated the model using the generalized method of moments approach of the Arellano-Bond estimator over a rolling window of 120 days [45-47]. The Arellano-Bond estimator offers several statistical advantages. It (1) enables a statistical examination of the model’s predictive ability and validity of model specification; (2) corrects for autocorrelation and heteroskedasticity; (3) is well suited for data with a small number of time periods and large number of countries; and (4) addresses omitted variables, providing a statistical test of correction validity. This method proved effective at identifying and statistically validating changes in the pandemic’s evolution within a period of 1 week. For a more comprehensive discussion of the method, see Oehmke et al [41,42].

To identify the appearance and duration of variants of concern, we also used data on sequenced SARS-CoV-2 variants from the Global Initiative on Sharing All Influenza Data (GISAID), which is an effective and trusted online resource for sharing genetic, clinical, and epidemiological COVID-19 data [48-51]. We used Nextclade nomenclature [52] to collect clade designations from sequences and Pangolin nomenclature for lineage designations of SARS-CoV-2 [53,54]. Metadata for the study period were collected on June 22, 2023. To avoid low frequency or potentially erroneous samples, the data set was further filtered to exclude months with fewer than 100 available samples, variant groups with fewer than 5 samples in a month, and variant groups representing less than 0.5% of the total samples in a month. The final data set consisted of 184,386 total samples available on GISAID [48-51]. All statistical analyses were conducted in R (version 4.2.1) with the *plm* package (version 2.6-2) [45,46]. For a snapshot of the data, see Multimedia Appendix 2.

We analyzed the potential “statistical end” to the pandemic with a 1-sided *t* test for whether the mean of speed was equal to or greater than the outbreak threshold of 10. We ran the test on a rolling 6-month window over weekly speed for the region, and we plotted the *P* values from the test over time.

### Ethical Considerations

This study followed the guidelines of the World Medical Association’s Declaration of Helsinki: Ethical Principles for Medical Research Involving Human Subjects [55,56]. This study was not submitted to the Northwestern University Institutional Review Board because all results are based on publicly available data with no private, identifiable information and the research did not involve any interaction with individual participants.

## Results

Table 1 presents the dynamic panel estimates for the most recent time window. The Wald test for the regression was significant (*P*<.001), and the Sargan test failed to reject the validity of the overidentification restrictions (*P*~1). Although the 1- and 7-day lag coefficients were statistically significant, suggesting a cluster effect in which cases on a given day impact cases 1 day and 7 days later, the coefficients were relatively small in magnitude (0.125 and 0.347, respectively). Furthermore, the shift parameters for either of the 2 most recent weeks were both significant and negative, meaning the clustering effect had become even smaller in the 2 weeks around May 5, 2023.

Traditional surveillance metrics for the weeks of April 28, 2023, and May 5, 2023, are provided in Tables 2 and 3. Except for Cyprus, every country had a small number of new COVID-19 cases. The next highest rate of new cases per 100,000 population was 3.04 in Georgia, considered a low transmission rate by the Centers for Disease Control and Prevention (CDC) [57]. This rate falls well below the informal threshold of 10 cases per day per 100,000 population [9,33-43]. Specifically, a “Low” transmission is considered no more than 10 cases per 100,000 population per week. “Moderate” transmission is 10 to 50 cases per 100,000 people per week. “Substantial” transmission is 50 to 100 cases per 100,000 people per week [57,58]. Although Cyprus appears to have been in a large outbreak in the week of April 28, 2023, the territory had 0 reported cases in OWID for the week of May 5, 2023. This drop most likely reflects episodic reporting, seen by several countries and territories around the world at the time.

Overall, the status of the pandemic around the WHO declaration in Central Asia is consistent with an “end” to the pandemic. An outbreak in Cyprus is restricted to the island. Based on the definition of a pandemic or an outbreak in several countries, the data indicate a shift from a pandemic to endemic COVID-19 in Central Asia, while it was epidemic in Cyprus. However, we note that metrics for Russia and Ukraine may be less reliable because of the ongoing war.

Comparing Tables 2 and 3 demonstrates little to no change before and after the WHO declared an end to the pandemic. Without question, Russia had the most cases of COVID-19 transmissions and deaths, but this rank is a function of population size. Thus, a better measure is the number of COVID-19 cases and deaths per 100,000 population. Moreover, death is often a better proxy for the state of an outbreak than transmissions because deaths are less likely to be undercounted [59]. Undercounting may be due to poor public health infrastructure, home antigen testing, or a dearth in polymerase chain reaction (PCR) testing or other resources. Azerbaijan and Russia each reported 0.02 deaths per 100,000 population. When we control for risk of death given the number of COVID-19 transmissions, we find that North Macedonia had the highest conditional death rate of 0.028 deaths per 100,000 population. Even though Cyprus was in an outbreak at the time the WHO declared the end of COVID-19 as a public health emergency, with 268 weekly cases per 100,000 population, the relative risk of death per infection was among the lowest in the region.

Tables 4 and 5 contain enhanced dynamic surveillance metrics for the 2 weeks before and after May 5, 2023. Speed was low for every country, and acceleration was either 0 or negative. The 7-day persistence effect on speed was also 0 or negative. These metrics suggest the pandemic may have indeed ended for the region. Because only a single country was in an outbreak, epidemiologically, COVID-19 would be considered an epidemic in Cyprus and not reach the threshold of a pandemic. We note that the figures in Tables 4 and 5 are calculated as day-over-day averages across the week. Thus, the magnitudes of speed, for example, tend to be roughly one-seventh the magnitudes of weekly speed in Tables 2 and 3.

Table 6 compares the 1-day persistence effect on speed for the 6 countries with nonnegative acceleration on the week of May 5 for both that week and the week prior. In each case, the effect was either 0 or negative and close to 0. Again, these metrics indicate that COVID-19 was well controlled in the region overall.

**Figure 1:**
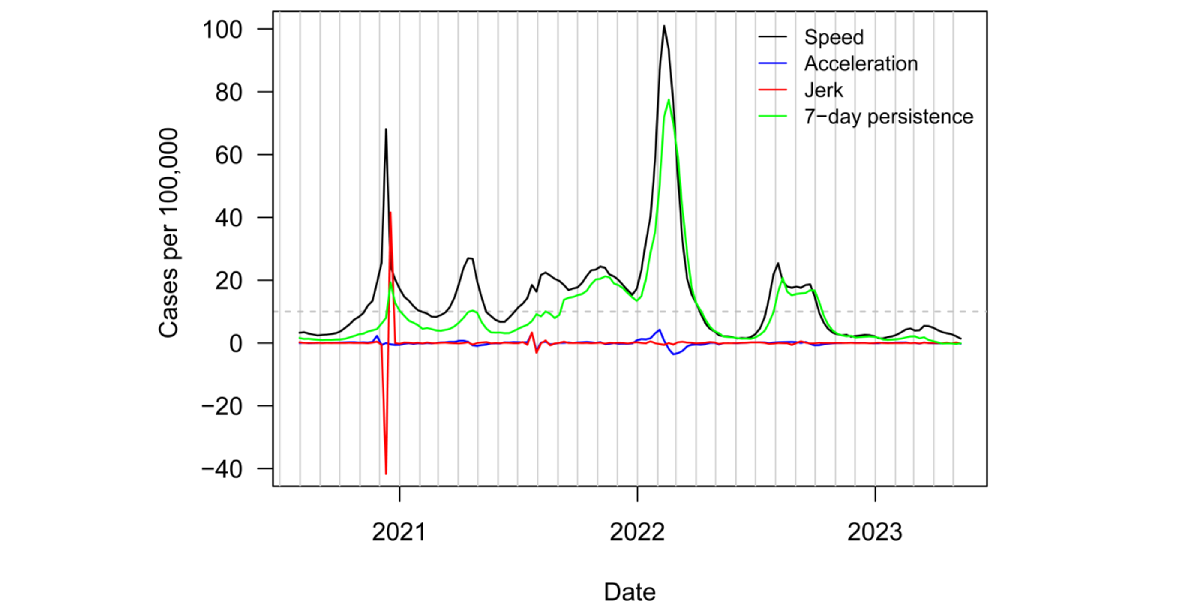
Enhanced weekly surveillance metrics (speed, acceleration, jerk, and 7-day persistence) for COVID-19 infections in Central Asia from July 31, 2020, to May 12, 2023.

Figure 1 plots regional speed, acceleration, jerk, and 7-day persistence metrics from July 31, 2020, to May 12, 2023. The horizontal, dashed grey line denotes the informal CDC outbreak threshold of speed equal to 10. The vertical, solid grey lines denote the start of each calendar month. The region was in a nearly continuous state of outbreak from November 2020 until April 2022. A final outbreak occurred from July 2022 to October 2022. The region saw a slight bump in cases around the end of February 2023. This bump occurred around the time of the Russian invasion of Ukraine. However, the region has since seen speed decline and remain stable around 2 new cases per 100,000 population.

**Figure 2:**
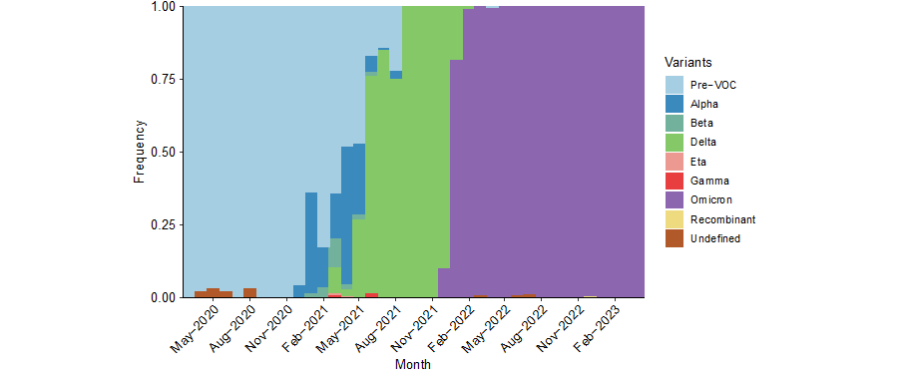
Variant groups as a proportion of all sequenced SARS-CoV-2 specimens from March 2020 to May 2023 in Central Asia (n=184,386). VOC: variant of concern.

Central Asia saw 2 pronounced outbreaks over the course of the pandemic. The first was a rapid spike in weekly speed, reaching a peak of 68 in December 2020. The second, even larger outbreak, reached a peak speed just over 100 in February of 2022. Figure 2 plots variant groups as a proportion of all viral specimens collected and sequenced in the region (and made available through GISAID) each month. The first outbreak occurred just around the appearance of the Alpha variant. The second outbreak was driven by the Omicron variant. Central Asia, like much of the rest of the world, saw a surge in cases amid the heightened transmissibility of Omicron [60].

Another potential indication of the end to the pandemic is the continued dominance of the Omicron variant. Although the region saw a mixture of the ancestral, Alpha, Beta, and Delta variants prior to the arrival of Omicron in November 2021, viral sequences have almost exclusively returned as Omicron and its subvariants ever since.

**Figure 3:**
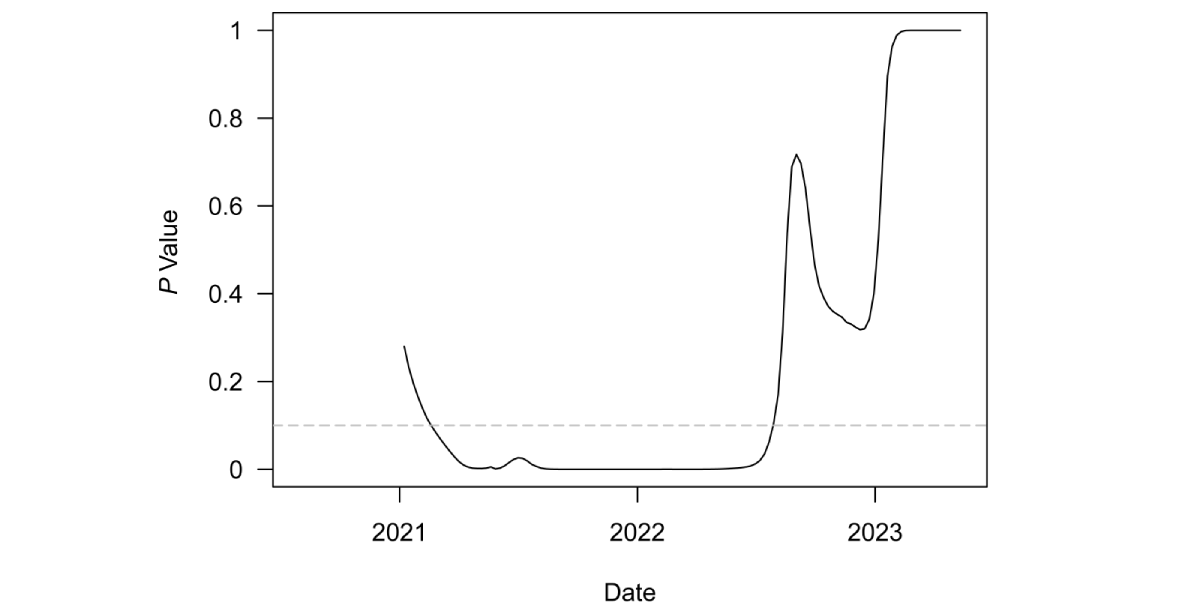
*P* values from *t* tests of weekly COVID-19 transmissions per 100,000 population equal to 10 over a rolling, 6-month window in Central Asia.

Figure 3 plots *P* values from a series of 1-sided *t* tests to determine whether speed for the region was equal to or greater than the threshold outbreak of 10. These tests were conducted on a rolling 6-month window of weekly regional speed. The dashed grey line denotes the least restrictive conventional significance level threshold of α=.10. The test first rejected the null in favor of the alternative for the 6-month period ending in mid-February 2021. From then on, the test rejected the null until the start of August 2022. The test statistic became insignificant from approximately mid-February 2023 onward. This more recent lack of statistical significance is consistent with the end to the pandemic in the region, as the test clearly failed to reject the null hypothesis of at least outbreak threshold speed.

With the historical context of enhanced surveillance metrics, the region appeared to be at the end stage of the pandemic. Speed had not been this low for this long since the start of the pandemic. We do note, however, that the military conflict in the region disrupted public health infrastructure. Data were likely to be underreported for Russia and Ukraine in particular. This reality brings some uncertainty to the ostensible end to the pandemic in the region.

**Figure 4:**
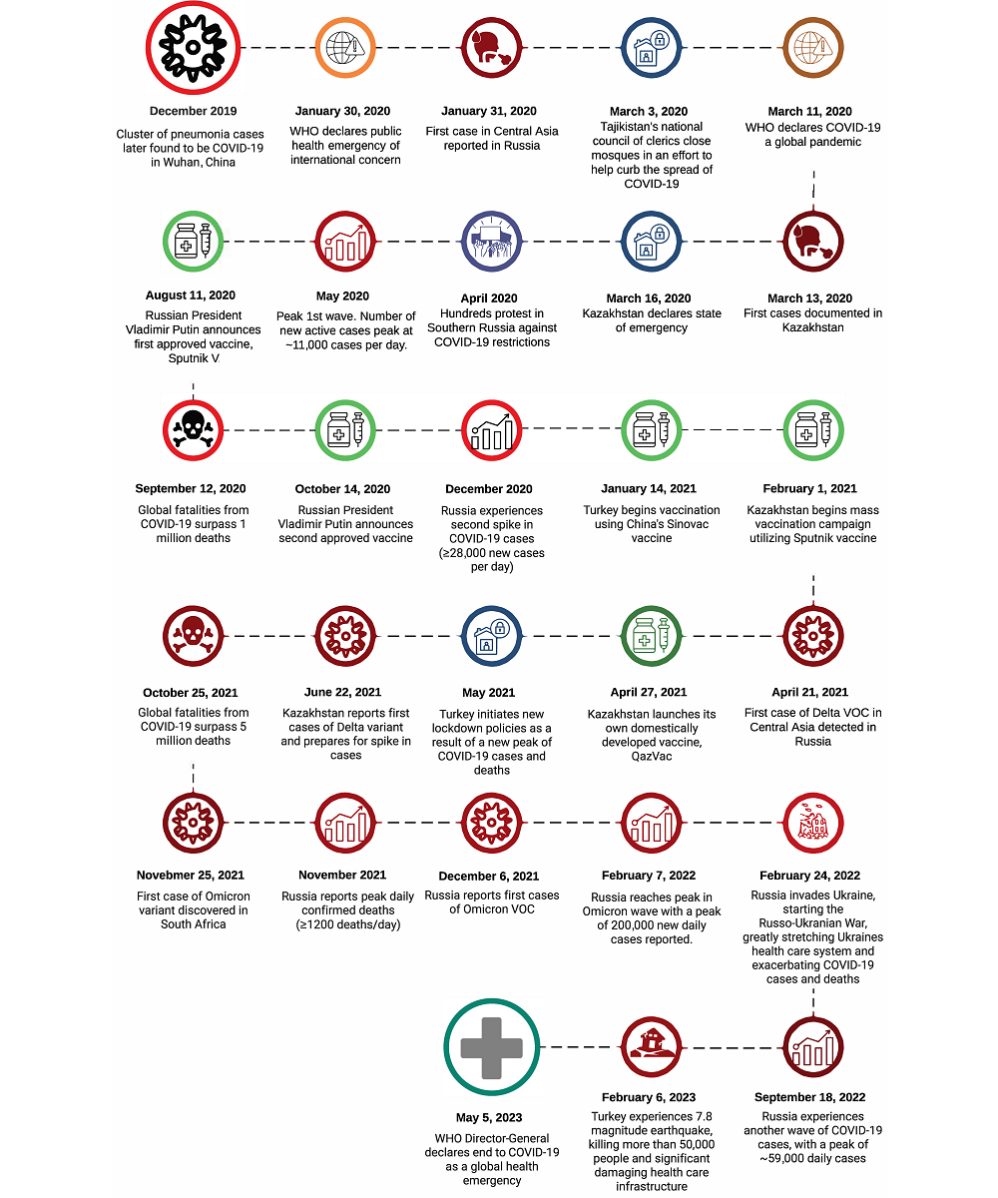
Timeline of the COVID-19 pandemic in Central Asia. WHO: World Health Organization.

Figure 4 provides a timeline of the onset of COVID-19 in Central Asia as well as vaccination programs and major events that likely created additional challenges to disease control, such as the Russian invasion of Ukraine and the earthquake in Turkey. Although Ukraine technically resides in Eastern Europe, millions of refugees fled Ukraine, accelerating the spread of disease in the region. Mass human migration is affiliated with increased disease transmission [61].

**Table 1:** Arellano-Bond dynamic panel data estimates of the number of daily COVID-19 infections reported by country in Central Asia from April 28, 2023, to May 12, 2023.

Variable	Value	*P* value^a^
1-day lag coefficient	0.125	<.001
7-day lag coefficient	0.347	.006
Shift parameter week of April 28	–0.385	<.001
Shift parameter week of May 5	–0.147	<.001
Weekend effect	–0.461	.60

^a^Wald test: χ^2^_6_=1641.02, *P*<.001; Sargan: χ^2^_540_=11, *P*~1.

**Table 2:** Traditional COVID-19 surveillance metrics for Central Asian countries in the week of April 28, 2023.

Country	New COVID-19 cases, n	Cumulative COVID-19 cases, n	New cases, 7-day moving average	Transmission (per 100,000 persons)	New deaths, n	Cumulative deaths, n	Death rate, 7-day moving average	Death rate (per 100,000 persons)	Conditional death rate
Armenia	0	449,113	7.29	0	0	8749	0.29	0	0.019
Azerbaijan	22	831,482	30.71	0.21	4	10,254	2.71	0.04	0.012
Cyprus	2404	660,854	343.43	268.30	10	1364	1.43	1.12	0.002
Georgia	114	1,841,495	123.29	3.04	0	17,065	0.29	0	0.009
Gibraltar	0	20,550	0	0	0	113	0	0	0.005
Kazakhstan	63	1,502,857	49.43	0.32	0	19,072	0	0	0.012
Kosovo	4	273,861	3.14	0.22	0	3206	0	0	0.012
Kyrgyzstan	0	206,888	1.71	0	0	2991	0	0	0.014
North Macedonia	0	348,215	10.43	0	0	9676	0.43	0	0.028
Russia	4215	22,870,557	4382.71	2.91	32	398,463	32	0.02	0.017
Uzbekistan	46	253,146	43.57	0.13	0	1637	0	0	0.006

**Table 3:** Traditional COVID-19 surveillance metrics for Central Asian countries in the week of May 5, 2023.

Country	New COVID-19 cases, n	Cumulative COVID-19 cases, n	New cases, 7-day moving average	Transmission rate (per 100,000 persons)	New deaths, n	Cumulative deaths, n	Death rate, 7-day moving average	Death rate (per 100,000 persons)	Conditional death rate
Armenia	0	449,148	5	0	0	8749	0	0	0.019
Azerbaijan	20	831,619	19.57	0.19	2	10,262	1.14	0.02	0.012
Cyprus	0	660,854	0	0	0	1364	0	0	0.002
Georgia	0	1,842,046	78.71	0	0	17,070	0.71	0	0.009
Gibraltar	0	20,550	0	0	0	113	0	0	0.005
Kazakhstan	0	1,502,857	0	0	0	19,072	0	0	0.013
Kosovo	0	273,876	2.14	0	0	3206	0	0	0.012
Kyrgyzstan	0	206,888	0	0	0	2991	0	0	0.014
North Macedonia	0	348,276	8.71	0	0	9677	0.14	0	0.028
Russia	3111	22,892,353	3113.71	2.15	28	398,658	27.86	0.02	0.017
Uzbekistan	42	253,405	37	0.12	0	1637	0	0	0.006

**Table 4:** Enhanced surveillance metrics for Central Asian countries in the week of April 28, 2023.

Country	Speed	Acceleration	Jerk	7-day persistence effect on speed
Armenia	0.26	0	0	–0.04
Azerbaijan	0.30	–0.06	–0.03	–0.03
Cyprus	38.33	38.33	38.33	0
Georgia	3.29	–0.06	0.08	–0.43
Gibraltar	0	0	0	0
Kazakhstan	0.25	0	0.01	–0.03
Kosovo	0.18	–0.03	–0.01	–0.03
Kyrgyzstan	0.03	0	0	0
North Macedonia	0.50	0	0	–0.06
Russia	3.03	–0.23	–0.01	–0.45
Uzbekistan	0.13	0	0	–0.01

**Table 5:** Enhanced surveillance metrics for Central Asian countries in the week of May 5, 2023.

Country	Speed	Acceleration	Jerk	7-day persistence effect on speed
Armenia	0.18	0	0	–0.01
Azerbaijan	0.19	0	0.05	–0.01
Cyprus	0	–38.33	–38.33	–1.44
Georgia	2.10	–0.43	0.03	–0.12
Gibraltar	0	0	0	0
Kazakhstan	0	–0.05	–0.01	–0.01
Kosovo	0.12	–0.03	–0.02	–0.01
Kyrgyzstan	0	0	0	0
North Macedonia	0.42	0	0	–0.02
Russia	2.15	–0.11	0.08	–0.11
Uzbekistan	0.11	0	0	0

**Table 6:** Comparison of 1-day persistence in the 6 countries in Central Asia with positive (nonnegative) accelerations for the week of May 5, 2023.

Country	1-day persistence week of April 28	1-day persistence week of May 5
Armenia	–0.02	0
Azerbaijan	–0.03	0
Gibraltar	0	0
Kyrgyzstan	0	0
North Macedonia	0	–0.01
Uzbekistan	–0.01	0

## Discussion

### Summary

The first aim of this study was to assess the status of the pandemic in Central Asia when WHO declared the end of the COVID-19 pandemic as a public health emergency of international concern. In line with the declaration, surveillance metrics suggest the COVID-19 pandemic had ended for the region and switched to an endemic. This categorization aligns with the distinction between pandemic and endemic occurrences, with the former characterized by heightened transmission rates and widespread disease propagation across an entire region. Conversely, the latter denotes the perpetual existence of a disease within a defined global area, devoid of abrupt surges in case numbers [13-16]. Still, to situate the period of the WHO declaration within the broader history, the COVID-19 pandemic had a substantial impact on Central Asia. As seen from Figure 1, the region was in a nearly continuous state of outbreak for most of the pandemic. For the 11 countries represented in Table 3, nearly 500,000 residents had died from COVID-19 by the time of the WHO declaration.

The war between Russia and Ukraine brought a bump in cases, which may be underestimated due to public health infrastructure damage in Ukraine and war propaganda in Russia. Despite the bump, the rate of new cases had been low and relatively stable for approximately 7 months by May 5, 2023. Another mass disaster that presented a COVID-19 challenge to the Central Asian region was the earthquake in Turkey in February 2023 that killed more than 50,000 people, rendering the public health infrastructure severely compromised. Mass disasters and large population movement are affiliated with disease transmission [62-67].

The impact of the pandemic in the region was driven partly by the region's reliance on extractive-sector exports and migrant remittances [68,69]. The closure of international borders exacerbated the region’s economic issues, including an undiversified and informal structure of production and exports, limited private sector involvement, and widespread employment gaps [68,69]. The economic contraction varied dramatically in the region, with Kyrgyzstan experiencing a 12.6% contraction, in contrast to Turkmenistan's reported 1.8% growth [68,70].

The Omicron surge in early 2022 had produced an all-time high in COVID-19 transmission rates when Russia invaded Ukraine in February 2023 [71-73]. The war also exacerbated the impact of the pandemic [74,75], as only one-third of the adult population in Ukraine had been fully vaccinated [76]. The war triggered the displacement of a significant portion of Ukrainians to other parts of Central Asia (as well as to other global regions), leading to surges in not only COVID-19 but other infectious diseases, including tuberculosis [71,72,77]. The conflict also disrupted access to critical medical services and created challenges for COVID-19 vaccine dissemination [71,72,77-80]. The lack of data reporting from the Russian government further fueled public health distrust both domestically and internationally [81-86].

Strict lockdown measures in Central Asia initially helped contain the spread of COVID-19, but the region’s public health care systems were eventually overwhelmed due to insufficient resources [87]. Countries in the region responded idiosyncratically to the pandemic. Some countries promptly acknowledged the virus and implemented containment measures, while others denied its existence and took limited action [68,87]. Russia's response focused on limiting contact with China, including closing borders and implementing strict quarantine measures [88,89]. However, some actions were criticized for being excessively restrictive [90,91].

Public health systems in Central Asia continue to struggle with high rates of infectious and chronic diseases [92,93]. Moreover, underfunding and corruption have resulted in limited access to quality health care in the region [68,94]. Although lockdown measures helped mitigate the impact of COVID-19, many countries in Central Asia experienced high morbidity and mortality [68,94]. Notably, North Macedonia and Georgia had the highest COVID-19 mortality rates in the region and ranked in the top 10 globally [95]. The pandemic also led to reduced health care services for other diseases like HIV, hepatitis, and tuberculosis, further adding to the public health burden [87]. Other humanitarian issues, such as the effects of climate change on agriculture and the Russian invasion of Ukraine, have posed unique energy and economic challenges to neighboring countries [68,96]. The slow recovery from the pandemic is attributed to the lack of reliable public health infrastructure, war, widespread poverty, and export-focused economies in the region [68,96-98]. As of May 2023, long-term financial and economic repercussions are still evident [99].

COVID-19 vaccine hesitancy was particularly high in Central Asia [100]. The development and acceptance of vaccines varied in the region. The early approval of Russia's Sputnik V vaccine drew criticism for its premature release without adequate clinical trials [101-107]. Despite widespread distribution, a considerable proportion of Russians remained hesitant to vaccinate [108-110]. Efforts to increase vaccine access in Central Asia involved partnerships with international organizations, such as the European Union and the WHO [111-115]. However, vaccination rates in the region still lagged behind more developed regions [116].

Economic policies in Central Asia aimed to augment social safety nets and support businesses during the pandemic [68,117]. Still, protests regarding the social toll of the pandemic emerged across the region [118]. In Cyprus, protests against lockdown measures and government corruption led to confrontations with the police [119]. Similar demonstrations occurred in Turkey in response to vaccination mandates [120]. In Russia, rallies and virtual protests criticized lockdown measures and the government's pandemic response [121,122].

Overall, the COVID-19 pandemic had far-reaching effects on Central Asia, impacting health care systems, economies, and social well-being. The region continues to grapple with the aftermath of the pandemic, and addressing its long-term consequences remains a significant challenge. Many countries in the region continue to face challenges due to limited economic and health care resources [9,93].

### Limitations

COVID-19 data had become less frequently reported around the world by the time the WHO declared an end to the pandemic [123]. Additionally, more people began to use at-home tests as the pandemic evolved [124], and the Russian invasion of Ukraine damaged public health infrastructure, which may have reduced the accuracy of reported cases in the region. The 7-day persistence measure is intended to mitigate the limitation. The model includes country fixed effects, which control for time-invariant, unobserved heterogeneity among countries. The estimates are also based on a rolling 120-day window, which limits the influence of changes in data reports outside of any particular window. Still, to the extent that a nonincluded country is unrepresentative of the region in disease burden, the omission of a country or territory can influence historical data comparisons. Viral specimen tests for variants of concern in GISAID are also dependent on testing and sequencing capacity, which varied by country across the region.

### Conclusions

The concern about potential resurgences of the virus is valid. As long as COVID-19 continues to spread and mutate, the possibility of new variants emerging remains. Variants could potentially be more transmissible, be resistant to vaccines, or cause more severe illness. This underlines the importance of continued vigilance, vaccination efforts, and global cooperation to control the spread of the virus [40].

Central Asia has experienced a relatively high disease burden from COVID-19, with approximately 500,000 deaths. For future pandemics, the ability to limit disease burden ahead of vaccines and treatment modalities will be a challenge, but the challenge can be informed and mitigated from the lessons of the COVID-19 pandemic. An epidemiological task force with a contact-tracing system, coupled with widespread testing of individuals, may be the first line of defense [125]. Lockdown policies, while costly, have also proven effective [126].

Although regional and political cooperation has been a bright spot for pandemic readiness around the world, including Central Asia, the region may face unique difficulties in a future pandemic if military conflict continues to pose a threat to public health [69,127]. Human migration caused by displacement, for example, is affiliated with increased disease transmission [61]. Regional and global efforts to promote peace are therefore an important tool for pandemic preparedness and response. Novel indicators of preparedness at the regional level could be helpful in these efforts, as they can identify countries in relative need of support [128]. Ongoing cooperation will be critical to reduce the disease burden of future pandemics, especially if a novel pathogen arrives outside of peacetime [129,130].

## Appendix

Multimedia Appendix 1 Glossary.

Multimedia Appendix 2 Data snapshot.

## Abbreviations

CDC: Centers for Disease Control and Prevention
GISAID: Global Initiative on Sharing All Influenza Data
OWID: Our World in Data
PCR: polymerase chain reaction
WHO: World Health Organization

## Data Availability

All data in this study are unrestricted and publicly available in the Our World in Data (OWID) and Global Initiative on Sharing All Influenza Data (GISAID) repositories noted in the Methods section [48,44]. All genome sequences and associated metadata are published in GISAID’s EpiCoV database. To view the contributors of each individual sequence with details such as accession number, virus name, collection date, originating lab, submitting lab, and list of authors, visit [131].
