# Supplementary material for: Surveillance Metrics and History of the COVID-19 Pandemic in Central Asia: Updated Epidemiological Assessment
Source: JMIR Public Health Surveill. 2024 Aug 28;10:e52318. doi: 10.2196/52318 (PMC11391161; doi:10.2196/52318)
Supplement: Multimedia Appendix 1 [file publichealth_v10i1e52318_app1.docx]

**GLOSSARY**

This list defines various epidemiological and surveillance terms used in this study:

*Speed of spread*: the rate of new COVID-19 cases per 100,000 population over a given time interval.

*Acceleration*: the difference in speed of spread from one time interval to the next, which identifies whether the number of new cases is increasing (positive acceleration), decreasing (negative), or at a stable inflection point (zero).

*Jerk*: the change in acceleration from one time interval to the next. Its name is adopted from physics nomenclature. A positive jerk can indicate explosive growth in the spread of a disease.

*7-Day persistence*: the statistical impact of 7-day lagged speed on current speed, which captures the predictive effect of cases at a given time on cases 7 days later.

*One-sided t-test*: statistical test for whether the mean speed of spread is significantly above the outbreak threshold of 10 new COVID-19 cases per 100,000 population over a six-month period.

*Cubic spline*: a statistical function used to assess the “smoothness” of data points and estimate missing or unclear data on a line given surrounding trends.

*Nextclade nomenclature*: an open-source tool for viral genome analysis, mutation identification, clade assignment, and phylogenetic mapping.

*Pangolin nomenclature*: an open-source tool (Phylogenetic Assignment of Named Global Outbreak Lineages) used to track the transmission and spread of SARS-CoV-2 and its lineages.
