# Supplementary material for: Surveillance Metrics and History of the COVID-19 Pandemic in Central Asia: Updated Epidemiological Assessment
Source: JMIR Public Health Surveill. 2024 Aug 28;10:e52318. doi: 10.2196/52318 (PMC11391161; doi:10.2196/52318)
Supplement: Multimedia Appendix 2 [file publichealth_v10i1e52318_app2.docx]

**Data Snapshot**

- EPI_SET_230801by is composed of 184,386 individual genome sequences.
- The collection dates range from 2020-03-01 to 2023-04-30.
- Data were collected in 14 countries and territories.
- All sequences in this data set are compared relative to hCoV-19/Wuhan/WIV04/2019 (WIV04), the official reference sequence used by GISAID (EPI_ISL_402124). Learn more at https://gisaid.org/WIV04.
